# Implementation of India’s largest sentinel surveillance for meningitis: an exploratory qualitative analysis of the facilitators and challenges in this sentinel site network

**DOI:** 10.3389/fpubh.2026.1731691

**Published:** 2026-02-16

**Authors:** Leyanna Susan George, Anurupa Chakraborty, Swati Gupta, Nivedita Gupta, Meningitis Surveillance Group

**Affiliations:** Indian Council of Medical Research, New Delhi, India

**Keywords:** meningitis, pneumococcal conjugate vaccine, qualitative study, sentinel surveillance, *Streptococcus pneumoniae*

## Abstract

**Background:**

Meningitis remains a leading cause of childhood morbidity and mortality in India, with *Streptococcus pneumoniae* as the most common bacterial pathogen. The Pneumococcal Conjugate Vaccine (PCV) was introduced into India’s Universal Immunization Program in 2017 with PCV-13 across five states and was later replaced by the indigenously manufactured PCV-10 in 2019. However, the absence of a nationwide surveillance platform for pneumococcal and other meningitis-causing pathogens limited data on disease burden and vaccine impact. To address this, the ICMR launched a project in 2018 to strengthen laboratory surveillance for pneumococcal meningitis, establishing India’s largest meningitis surveillance network. This paper documents the challenges encountered during its implementation and the actions taken to overcome them, offering insights for countries planning similar surveillance systems.

**Methods:**

An exploratory qualitative evaluation was conducted to identify facilitators and challenges faced during implementation of the surveillance network. Using multistage purposive sampling, 15 study sites across six zones of India were selected, representing National Apex Laboratories, Regional Reference Laboratories, and Clinical Recruitment Sites. Twenty-five key informant interviews were conducted with project implementers from both high- and low-performing sites. Audio-recorded data were transcribed, translated, and analyzed using a framework approach.

**Findings:**

The project team encountered challenges throughout the implementation of the surveillance network, including case identification, obtaining informed consent—particularly from illiterate populations—and incomplete clinical or vaccination histories. Difficulties were also noted in CSF collection, storage, transport, and delayed laboratory reporting. Data management issues arose from manual CRF entry, along with interdepartmental coordination problems due to frequent staff transfers and the need for repeated training. Dependence on external supplies for reagents and primers posed additional limitations. These challenges were addressed through root cause analysis, timely corrective and preventive actions, regular trainings, improved supervision, and enhanced communication via SOPs and digital platforms. Key facilitators for successful implementation included stakeholder motivation, capacity building, and strengthened coordination mechanisms.

**Interpretation:**

The successful implementation of this nationwide laboratory surveillance despite multiple challenges offers valuable lessons for future initiatives. A decentralized sentinel laboratory network with integrated data management and strong central coordination is crucial for effective monitoring. Based on this experience, establishing similar surveillance systems is recommended for countries to track circulating pneumococcal serotypes and support evidence-based vaccine decisions.

## Introduction

Meningitis is an acute and potentially life-threatening condition that involves inflammation of the meninges, and it can be caused due to various infectious agents, primarily bacteria, viruses, and fungi. Among these, bacterial meningitis is particularly severe and demands urgent medical intervention to prevent long-term complications, such as neurological impairment, sensory deficits, and even death (1).

The burden of meningitis disproportionately affects low and middle-income countries (LMICs), where access to healthcare resources and vaccination programs are often limited (2). Studies indicate that *Streptococcus pneumoniae*, *Haemophilus influenzae* type b (Hib), and *Neisseria meningitidis* are the primary bacterial pathogens responsible for meningitis, particularly in LMIC regions. These pathogens contribute substantially to morbidity and mortality rates, especially among children under 5 years of age (3). In India, *Streptococcus pneumoniae* has been identified as the leading cause of bacterial meningitis in hospitalized children less than 5 years of age, accounting for approximately 82.9% of confirmed cases. The second most common pathogen, Hib, accounts for around 14.4% of cases, followed by *Neisseria meningitidis* at 2.7% (4).

Vaccination is the most effective strategy for countries to control infectious diseases (5). Vaccines against Hib, *Streptococcus pneumoniae*, and *Neisseria meningitidis* have significantly reduced bacterial meningitis in children globally. Many National Immunization Programs (NIPs) have adopted vaccines against these pathogens thereby greatly reducing the disease burden (6). The Hib vaccine is a part of the pentavalent vaccine and is available in over 193 countries, including India (7). The Pentavalent vaccine was introduced in a phased manner in India in 2011 and was initially launched in Tamil Nadu and Kerala, followed by the introduction in the other states (8). In 2015, The National Technical Advisory Group on Immunization (NTAGI), India recommended that a pneumococcal conjugate vaccine (PCV) be introduced into the Universal Immunization Program (UIP) (9). PCV-13 was introduced in a phased manner across 5 states in 2017 with plans for introduction in the other states. The roll-out was delayed due to the COVID-19 pandemic that affected the supply chain and staffing. However, irrespective of these challenges India successfully rolled out the first domestically produced PCV-10 to the entire nation during the ongoing pandemic in 2021 (10).

Prior to introduction of PCV in India, the country lacked a dedicated, nationwide surveillance platform for pneumococcal and other meningitis-causing pathogens, limiting the availability of data on the disease prevalence and the effectiveness of vaccination programs. In this context, the Indian Council of Medical Research (ICMR), in collaboration with the Gates Foundation, launched a project titled the “Strengthening Laboratory Surveillance for Pneumococcal Meningitis in India” in the year 2018. The aim of the study was to evaluate the impact of the PCV rollout on pneumococcal disease burden and to gather comprehensive data on circulating *Streptococcus pneumoniae* serotypes across the country. To achieve these aims, a meningitis surveillance network was launched which comprised sentinel sites with national representation (11). It was designed as a three-tiered hierarchical system. The lowest tier consists of 33 Clinical Recruitment Sites (CRS) which are tertiary level hospitals like medical colleges where children less than 5 years of age suspected of meningitis are being admitted in the Pediatric ICUs/ Pediatric neurology departments. Pediatricians from these departments identify and recruit participants. After obtaining informed consent from the parents of the children, clinical, demographic and vaccination data are being retrieved. Cerebrospinal fluid (CSF) samples are also being collected and sent to the microbiology lab of the tertiary centers for further processing and storage. These samples are then transported to the Regional Reference Laboratories (RRLs) which are the second level in the three-tier surveillance system. There are four RRLs which are tertiary care centers and ICMR institutes that are responsible for the testing of the CSF specimens for *Streptococcus pneumoniae*, *Neisseria meningitidis* and *Haemophilus influenzae*. Each of the RRLs is linked to 4–6 CRS, to monitor their progress and conduct the lab assays on the CSF samples collected by them. The top tier consists of two National Apex Laboratories (NALs), each equipped to conduct advanced molecular diagnostics. Each NAL is linked to two RRLs and are carrying out *S. pneumoniae* serotyping using Taqman Array Card on the samples transported to them from RRLs after testing positive for *S. pneumoniae*. The serotyping data of the circulating pneumococcal strains helps to assess the vaccine efficacy. The data is managed by ICMR—National Institute of Epidemiology (NIE) which also functions as an RRL. While the overall implementation and monitoring of this sentinel surveillance network is being carried out by the ICMR Headquarter at Delhi, India. This was the first time ever a nationally representative meningitis sentinel surveillance network has been established in the country that spans across all 6 zones of India.

The implementation of this robust surveillance system for meningitis, presented with several challenges. Effective surveillance requires substantial financial investment, trained personnel, and adequate infrastructure, all of which can be limited in resource-constrained regions. Additionally, data collection for meningitis cases is complicated by underreporting, lack of vaccination data, especially in rural and underserved areas, where access to healthcare facilities and diagnostic tools are often limited. Addressing these challenges necessitated a multifaceted approach, involving the collaboration, coordination and effective communication between different stakeholders.

This project was launched by ICMR in collaboration with Gates foundation in the year 2018 for a duration of 5 years. However, it was fully initiated only in 2019 following the obtainment of all clearances. The project did face a setback due to the COVID-19, however it continued functioning during the pandemic as feasible and was fully functional post the pandemic. The project was initially planned to conclude in June 2023 and in this regard it was decided to conduct an exploratory qualitative analysis in order to document the facilitators and challenges faced in the implementation of this surveillance network. This qualitative evaluation was conducted from March to June 2023, however transcription, data analysis and report preparation was conducted from October 2023 to March 2024. Due to the setback faced by COVID-19, the project has been granted a no-cost extension till June 2026, following which it is planned to be integrated with the routine surveillance network of Infectious Disease Research and Diagnostic Laboratory.

Therefore, the objective of this analysis was to conduct an exploratory qualitative evaluation of the experiences of the stakeholders involved in the implementation of this meningitis surveillance network. This analysis seeks to offer insights into the complexities of establishing a nationally representative sentinel meningitis surveillance system by identifying the facilitators and challenges faced by the stakeholders and also documents the strategies that were put in place to establish this network.

## Methodology

In order to capture a holistic over view of the facilitators and challenges faced in the implementation of the meningitis surveillance network, the study participants were selected from all the three tiers using multi-stage purposive sampling. This qualitative evaluation was conducted toward the end of the project period in order to capture the challenges experienced throughout the different phases of project implementation. Since there are only 2 NALs each responsible for carrying out advanced molecular tests on samples and there are 4 RRLs that are overseeing the activities of the CRSs all these sites were purposively selected for this evaluation. Out of the 33 CRS, 8 of them were selected in consultation with the Principal Investigators of the RRLs who were responsible for overseeing the activities of the CRS. In order to get the perspectives of both the good and poor performing CRSs, all the CRSs were initially grouped based on their location across the 6 zones of India. From each of the 6 zones, the Principal Investigators of the RRLs responsible for the CRS allotted to them were asked to classify the CRS based on their performance. Multiple indicators were used to assess the performance of the CRS such as number of suspected cases of meningitis, in patient admission of meningitis cases and collection of CSF samples, confirmed cases of *Streptococcus pneumonia* at the CRS sites. In addition, other factors that were crucial for successful project implementation such as obtainment of participant informed consent forms, vaccination data retrieval, sample collection and transportation, data entry etc. were also considered. Based on these indicators, the PI’s of the RRLs classified the performance of the CRS as good and poor performers. Four CRS with high scores and four with the lowest scores were selected for this qualitative evaluation.

**Figure 1:**
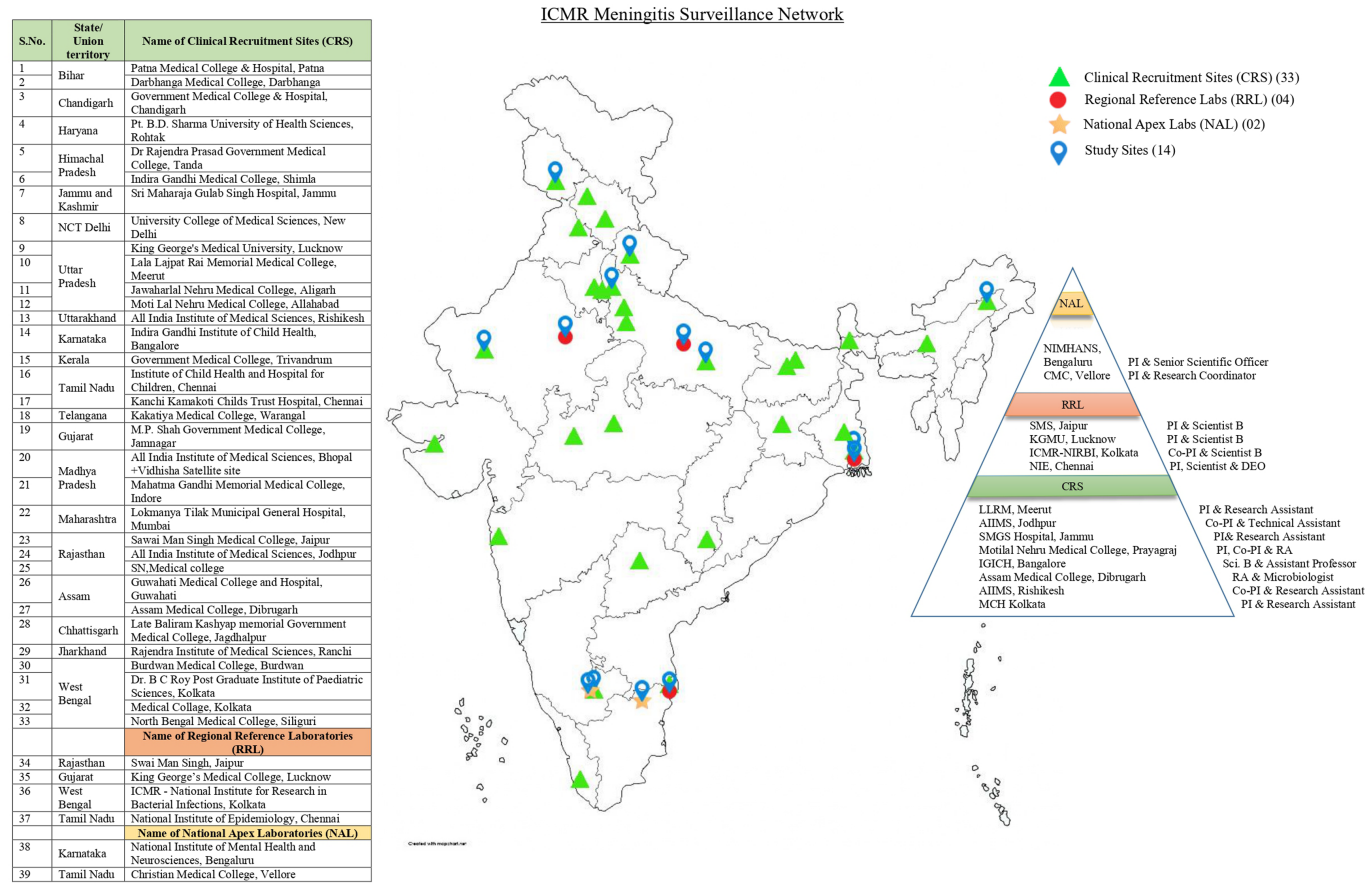
Map depicting the ICMR meningitis laboratory surveillance network sites.

At each selected site, the key informants were purposively selected based on the role they played in the project in order to capture the facilitators and challenges they faced at each stage, i.e., study participant identification, enrolment, data and sample collection, laboratory analysis, data entry and analysis. The study implementers who are the main stakeholders in the project were contacted and prior appointments were taken for conducting the interviews. The Key Informant Interviews (KIIs) were conducted after obtaining informed consent and permissions to record the interviews. The KIIs were conducted virtually using in depth interview guides which were developed after extensive formative research. The guides were developed keeping in mind the different activities that are being conducted in this surveillance network. Separate questions were prepared for each of the stakeholders based on the work being carried out by them so that a complete picture of the facilitators and challenges faced for setting up such a nationwide surveillance network can be captured. KIIs were conducted till data saturation was attained with no further information being shared. The details of the selected study sites and participants are shown in Figure 1.

The study was conducted by investigators trained in qualitative research from the Indian Council of Medical Research (ICMR) Headquarters. Approval of the institutional ethics committee was obtained from these institutes before study initiation.

## Analysis

All interviews were conducted in English, and the audio files were transcribed verbatim by the project team. The two investigators responsible for data analysis, then cross checked the transcribed data with the audio recordings to ensure that the data was completely captured with no deletions or additions, thereby ensuring quality of the transcribed data. Transcripts were then read and re-read multiple times in order to gain familiarity with the data. An inductive approach was used to code the data manually. The codes were entered into a common code book which was used by the two investigators responsible for data analysis. New codes were added as and when it appeared on reviewing the transcripts after discussion and agreement by both the investigators thereby maintaining the uniformity of the codes. Using this common code book, all transcripts were coded and emerging patterns in the form of themes and sub themes were identified and grouped together under specific domains. The grouped data in the form of quotes was then charted into a framework without losing the essence of the data. This charting exercise helped to summarize the data by category from each transcript and it also enabled a process of cross-comparison between the different interviews. This framework was independently reviewed by both the researchers to draw conclusions in order to increase the validity of the findings. The discrepancies that evolved were resolved through discussion till consensus was reached between the researchers. Conclusions were drawn by comparing and triangulating the data between and within the 2 NALs, 4 RRLs, 4 high and 4 low performing CRSs. This helped the researchers not only to identify similar patterns and to look into divergent findings but it also lead to achieving data saturation. Data and researcher triangulation methods enabled to increase the validity and reliability of the study findings.

## Results

**Figure 2:**
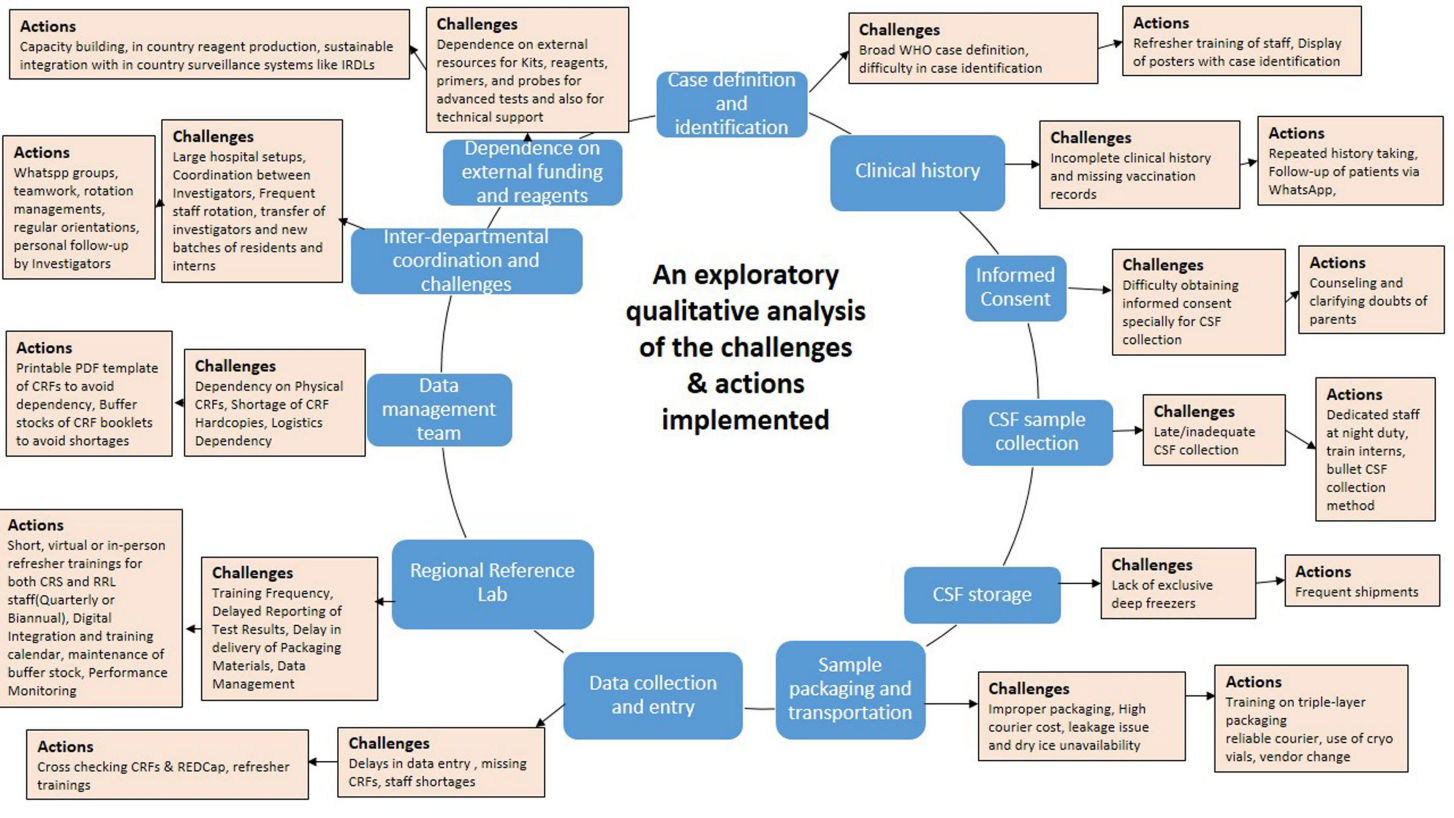
Summary chart depicting the major implementation challenges and actions implemented.

A total of 25 Key Informant Interviews (KIIs) were conducted among the project team members hence forth referred as study implementers that included Heads of Departments, Principal Investigators (PIs), Co-Principal Investigators (Co-PIs) who were either pediatricians or microbiologists, Scientists, Research Assistants (RAs), data managers, statisticians etc. These study implementers were spread across 14 different institutes that were representative of each of the three tiers of the surveillance network. The KIIs were conducted till data saturation was obtained with no new themes arising. A summary of the results of this qualitative exploratory analysis is depicted in Figure 2.

The KIIs with the different study implementers began with questions regarding their job responsibilities and work satisfaction. It was observed that all study implementers were well versed with their job responsibilities and were in general satisfied that they were contributing to a project of national importance. Following this, the participants were asked about the different steps in implementation of the surveillance network, the first being participant recruitment into the project.

### Case definition and identification

The most crucial step in this surveillance network was the identification of meningitis patients for which the WHO case definition was used (12). Most of the investigators felt that the clinical case definition was broad using words such as “lethargy” and “altered sensorium” which are less common presentations in children at the sentinel surveillance sites.

Although most respondents described difficulty identifying cases present at the study site, this was alleviated with refresher trainings. These trainings were also found to be effective for increasing patient enrolment since the staff were reminded about the study and new rotational staff were being continuously trained.

*“There are no challenges in case identification. At least there are 6 to 7 residents in the department right from SRs to JRs and now since it has been already four years, they know that this project is running, so they promptly take extra samples of CSF.” CRS PI who is the Professor and HOD of Paediatrics.*

One of the other challenges that the site Principal Investigators (PIs) faced for case identification was eliciting the clinical history of the patients making case identification difficult. This challenge was overcome by the Research Assistants spending more time with the patients in eliciting the clinical history from the parents.

*“Most of the patients are from villages so the history provided by them is not clear. The parents do-not even know how to check the fever of their child. They can only convey whether the fever is high or low but cannot say the exact temperature. They tell a different history to the doctor, but when I sit and discuss with them longer, I get all the details needed.” CRS Research Assistant, Paediatric Department.*

### Informed consent forms

Following the case identification, the next step in the study was to obtain the informed consent from the parents of the sick child for the enrolment in the study and also for collection of CSF sample. Obtaining consent posed difficulties in some rural areas where most of the parents were illiterate and they were hesitant to provide consent for CSF collection. These are exemplified by the quotes below.

*“Consent is not a problem. We get it easily from the patients. Sometimes the problem comes for the CSF. Patients do not give the consent for the lumbar puncture.” CRS PI, Professor and Head of the Department of Microbiology.*

*“They did not even agree to give their thumb impression, made excuses stating that their husbands would come and will give consent. But when the husband came, he did not agree. He said they will share all documents but will not give signatures or thumb impressions.” CRS, Technical Assistant.*

However, these challenges were overcome by properly counseling and clarifying the doubts of the parents.

*“We make the patients and family members understand about all the plus points of the study like the tests which will be done if they agree, some times they do not agree. Considering one or two patients out of 50, others all give consent.” CRS Research Assistant.*

Obtaining informed consent from parents of sick children was identified to be a challenge at some of the CRSs. This was observed to be a challenge especially among illiterate parents. Even though some of the parents agreed to share data, some were hesitant to provide CSF samples. In such cases, it was observed that the study implementers made all efforts to provide information regarding the study and its usefulness. Efforts were made to spend extra time with the parents to patiently clarify their doubts and concerns. If they were convinced to participate in the study and provide CSF then only they were included in the study. Children whose parents were unwilling even after counseling were excluded from the study but all their health care needs were addressed as required. Only after the parents provide informed consent for both sharing data and collecting CSF, then only the children were enrolled in the study. This was then followed by a thorough clinical history and examination of the child and filling the data in the case report forms.

### Clinical history

Obtaining adequate history of prior use of antibiotics and presence of co-morbidities in the sick children were found to be quite challenging because most of them come to hospital without any medical documents. Many of the sites tried to track this information by reaching out to the hospital that referred the patient. Additionally, parents were asked to retrieve their records and bring them during the follow up visits. They also tried to track the information using the unique health ID which was found to be time consuming with little yield.

*“Patients who get referred generally do not bring their discharge papers, or if they bring something it’s just the front page of the discharge paper in which the doctor referred them to that particular hospital…rarely it has any information regarding the antibiotics prescribed.” Technical Assistant, CRS.*

*“Most of the time patients cannot tell about antibiotic usage. Illiterate patients are unable to provide any information. Those who are literate can still give some information because they have the doctor’s prescription with them where they were admitted earlier” Research Assistant, CRS, Paediatric Department.*

Retrieval of vaccination history was challenging. Since most care takers/ patients often rushed to the hospital, and they did not carry vaccination cards with them. Information if PCV vaccine was given to the child and if yes, its composition whether it was PCV-10 or PCV-13 was crucial for understanding the serotype circulation in the country. Most parents were unaware and this information was difficult to retrieve. However, the site investigators made extra efforts to retrieve them by reminding them to send it via WhatsApp whenever they went home to collect materials or when they were discharged. However, some of them did not have smartphones and they were unable to take a picture of the vaccination card and share it with the health care workers.

*“We do not get the exact history of vaccination. Most of the time we do not get any vaccination card, we only get the information whether the child is vaccinated or not. Most of the patients’ attendants are illiterate and they even do not know whether their child has been vaccinated or not.” PI, Professor and Head of the Department of Microbiology, CRS.*

However, there was also a subset of participants who left against medical advice (LAMA) either after being admitted for a long time or the child not showing any signs of recovery. The sites all lacked information about what happened to the child after leaving the hospital, whether the child survived or died. This information was the most difficult to recover.

### CSF sample collection and storage

Following clinical examination and history taking, the next step in the project was to collect CSF samples from children. The study investigators were asked if they faced any issues regarding CSF sample collection, packaging, transportation and storage.

One of the major challenges faced in the collection of CSF was the timing of the patient reporting to the hospital. It was observed that at some sites, cases reported to the hospital in the evenings at which time research assistants were not available. Hence, for many of these patients, the CSF samples would be collected by inexperienced interns, many of which resulted in inadequate samples. For others, CSF samples were not obtained during the night shift and intravenous antibiotics would have been started before the CSF samples were collected in the morning or they would have been totally missed out.

*“Most of the paediatric meningitis cases reported in the evening and CSF did not get collected. Since the microbiology department timing is from 9 a.m. to 4 p.m. and research assistant is available only during that time. The samples collected after 4 p.m., had many problems such as CSF not collected properly, stored properly, insufficient amounts etc. Missing those cases can be a great loss since antibiotic treatment was already started before CSF collection” CRS PI, Professor and Head of the Department of Microbiology, LLRM Meerut.*

However, this challenge was overcome by the concerned department by appointing a dedicated and trained resident for this project who would be available for collecting the sample from patients reporting in the evening to the hospital. However, there are times, where the patient load was high and there was a shortage of staff resulting in delays of obtaining consent and CSF collection. As one of the PIs stated:

*“Numbers of admissions are more in the morning, and the staff is less so they may not be able to collect the CSF in the morning or on the same day and may be collecting it next day, so it definitely hampers the results” CRS PI, HOD Paediatrics.*

It was also observed that the decision to enroll a participant in the study and to collect their CSF was made by the pediatrician and his/her team. Even though, the microbiologist felt that the case could be enrolled, the pediatrician made the final decision based on the clinical relevance. As one microbiologist stated:

*“Particularly at our site the challenges we faced while recruiting the cases was that the discretion of doing LP was with the treating paediatrician. So we do not have 100% conversion of case recruitment into CSF examination.” CRS PI, Assistant Professor in Department of Paediatrics.*

This challenge was overcome by having the case definitions printed out as posters and being pasted in the wards, Additionally, training to all staff and students was reinforced regarding the need for the study and the importance of doing LP when they were not sure, thereby increasing the case recruitment and LP rates as well. Another motivating factor to increase LP was to provide feedback of positive pneumococcal samples. This encouraged the young interns and residents to continue doing LP for suspected cases in order to potentially confirm a diagnosis.

Another challenge observed was the presentation of cases following antibiotic treatment. Many cases presented to the tertiary care centers following a prolonged course and antibiotic therapy, making them ineligible to participate in the study.

*“Children reached us towards the end of the first week of care or towards the beginning of the second week of care. Most of the time they are partially treated or incompletely treated so we did not have clarity in recruiting them at that point of time. Also, immediate CSF examination is also not possible at that stage.” CRS PI, Assistant Professor in Department of Paediatrics.*

At some of the sites it was observed that even though CSF was collected, it was found to be of insufficient volumes. The reasons stated for this was the need to perform and prioritize routine diagnostic tests over study-specific tests. While at some sites, the CSF was collected for thesis work of the postgraduate students resulting in insufficient volumes. Inadequately trained new interns/residents also resulted in poor CSF tapping. Last but not the least, there were severely dehydrated patients hence CSF tapping often resulted in insufficient volumes. Below are some excerpts from the study personnel:

*“Sometimes, I do not get a sufficient volume of samples for the study. The samples are being send to 3 different labs -Pathology, Biochemistry & Microbiology for testing which lead to a shortage.” Technical Assistant, CRS.*

*“Many times, out of 200–300 samples you may get one or two samples with insufficient volume. Usually this happens whenever there is a new batch of residents. CRS PI, Additional Professor, Department of Paediatrics.*

The actions taken by the sites to rectify these issues were as follows:

One of the PIs stated*: “A few samples may be of inadequate volume, but we sorted out. I call the first-year residents personally and give orientation regarding the requirement of the project and usually things get sorted out. While doing LP If we get low volume then we prioritise to send it for diagnostic testing*.” *CRS PI, Additional Professor, Department of Paediatrics.*

As a part of the training, this site also carried out dummy rounds of sample collection to train the new residents on how much volume needs to be collected. As the PI stated: *“Initially we do a dummy measurement like take normal saline or distilled water in a syringe and put it in the vial, so they know this is the approximate volume that has to be sent, then they replicate it with CSF.”*

In order to overcome the insufficient CSF sample that needs to be redistributed to different labs, one site introduced a good practice of using “bullets” which are atraumatic needles with a blunt tip used for conducting lumbar puncture for CSF collection. The site investigators claimed that it not only decreased pain but also improved the quantity of the CSF collected.

*“Whenever a CSF sample is taken it needs to be sent to the pathology, biochemistry and microbiology departments for cytology, glucose and protein analysis, gram stain and culture. So initially the quantity used to be insufficient. So then we tried to introduce bullets in our LP. This way we ask them to collect CSF in 1 or 2 bullets separately. In one sample 0.5 or 0.8 mL can be preserved for microbiology purpose, and this way we started getting better quantity.” CRS PI, Assistant Professor in Department of Paediatrics.*

Some of the sites also stated that antibiotics were either given or started before reaching the study center. Hence CSF samples were not taken. If CSF was collected in the previous hospital and the results were available, they were recorded for patient treatment.

### CSF sample storage

Some sites did not have an exclusive deep freezer at their department for the study, and they had to share it with other departments, resulting in a space crunch problem. Hence, to overcome this issue frequent courier of samples to the RRLs were carried out at regular intervals.

In some sites, CSF samples were collected and stored in refrigerators before being sent to the labs and in the initial stages of the study, temporary storage of the CSF sample in refrigerators did lead to mishaps such as misplacements and loss of CSF samples.

*“Sometimes storage of samples is an issue. Samples are kept in the fridge with many other medicines. One time the samples got lost. There is no designated space provided for this project to keep CSF samples in the ICU and we need to share the same space. Another problem is we have a limited group D staff to carry the samples to the lab. We even used to ask the parents or local guardians of the patient to take the samples to the different departments. Sometimes they used to misplace or lose the sample.” Co PI, Professor and HOD of Department of Paediatrics, Medical College, Kolkata.*

In the satellite sites, where CSF was collected, the samples were stored and transported in vaccine carriers with ice packs to the labs and were then stored in deep freezers. Thereby maintaining the cold chain all the time. Another challenge observed was the lack of proper packaging in some laboratories. When such issues were observed, SOPs for proper sample packaging were shared with all sites and they were trained regarding triple packaging.

*“We have received a SOP from NIE regarding packaging. First of all, we take the volume and put it into the cryovial then we do the triple layer packing before shipping the batch to NIE” Scientist, CRS.*

### Sample transportation

The samples collected at the CRS need to be transported from the CRS to RRLs. At CRS sites that were near to RRLs, the research staff used to pack the samples and would deliver them in person. However, in the remote sites, the RRLs often would arrange a courier service that would collect the samples, pack them, and deliver to the respective RRLs. All CRS sites using these services found the courier service to be reliable.

*“We have the same courier named ‘track one’ that is working with us for 4 years so it’s reliable” Research Assistant, Department of Microbiology.*

However, in some sites the research assistants had to travel quite a distance either by bus or train to reach the main city where the courier service was present. As stated by one PI:

*“Most of the times we send a person, our data entry operator, he goes once the sample count goes up to 100. He goes by train from Bangalore to Chennai which takes around 5–6 h.” Scientist B, CRS.*

The courier service also did triple packaging of the samples and transported them in dry ice to the RRLs.

*“Sample transportation is not a problem. ICMR itself has told us about Tempcon express. We gave him the samples. He packs the samples in dry ice”- Research Assistant, CRS.*

The courier costs varied across sites based on distance. In some sites, the cost was as low as Rs.140–150 per packet. However, at the other sites the cost was high because of the dry ice.

*“The Dry Ice costs us more than Rs 10,000/− to Rs12, 000/− and we have to give advance for buying since it is not available most of the time” Technical Assistant, CRS.*

In places where dry ice was not available it had to be brought from elsewhere and it had to be transported resulting in an increase in the cost of the courier.

*“Dry ice is not available in Meerut so it has to be purchased from Noida which is very costly. There is nobody to deliver it and hence cost of courier also gets added” CRS PI, Professor and Head in the Department of Microbiology.*

It was also seen that post COVID-19 pandemic, the cost of dry ice increased due to increased demand for shipping of samples for testing across the country. As one of the investigators stated:

*“Before COVID the courier person was charging Rs 13,000 but post COVID he is charging around Rs18,000*.” *Research Assistant, CRS.*

All sites were extremely careful to avoid any leakages, so they packed their samples well by doing triple packaging.

*“There is triple packaging. Samples in cryo vials are kept in a plastic container and then packed along with ice packs in a thermocol box. Each cryo vial is surrounded by either cotton or absorbent like tissue paper.” CRS PI, Professor and Head of the Department of Microbiology.*

However, it was observed that in some sites the leakages were not caused due to faulty packaging or transportation but due to the poor quality of the vials in which the samples were stored. As stated by a research associate:

*“There was a leakage issue because of the quality of vials we were using.” Research Assistant, CRS.*

However, corrective measures were taken promptly, and the vendor was changed. Thereby ensuring the provision of superior quality vials.

Once, the samples were received at the RRLs and they were analyzed, the data was entered in the ReDCap.

### Data collection and entry

At all sites, data was entered first into the source documents, i.e., the hard copies of the eCRFs. It was then entered into ReDCap, the data management software at the sites. The hard copies were then shipped to the data management team who did the quality check of the data in ReDCap before it was locked and analyzed. There were minor challenges faced for data collection such as missing data in the source documents. At one site, one of the PI’s stated that *“Sometimes there is a challenge when some data is missing. Sometimes the data have been filled in the ReDCap portal but not in the hard copy” CRS PI, Professor and Head of the Department of Microbiology.* However, all such data was cross-verified by the data management team before analysis.

Most of them easily understood the questions in the CRFs, however, some questions were found to be confusing for some of the staff. As a CRS PI, Assistant Professor in Department of Paediatrics stated: *“When collecting data, there used to be grey areas for our nursing staff and project scientists when they wanted to fill up the data for immunization history or the chronology of symptoms in such situations.”* In such situations, these queries were resolved during the monthly project review meeting, and it was followed by a refresher training of all staff members by the data management team.

Getting information on certain questions was challenging. Parents often did not remember the vaccination details or have the vaccination card, hence this data was found to be often missing in the CRFs. Use of antibiotics, its name and dosage given were often not recorded if the parents did not come with a discharge history or prescription. Also, the timing of antibiotics given with respect to the timing of lumbar puncture for CSF collection was crucial information which was found missing in some cases. “*There is a question no. 33 in CRF asking whether antibiotics were given before or after LP. So, if it was taken, the timing was unknown whether it was more than 4 h or not between LP and antibiotic.” Technical Assistant, CRS.*

Other challenges faced at the sites were insufficient hard copies of the CRFs which then had to be photocopied. Non-availability of data entry operators or computers exclusively for the study were seen to cause delays in data entry at some sites.

### Challenges with RRL

Each of the four RRLs was allotted 3–5 CRS from where samples are routinely being collected and sent to the RRLs for laboratory analysis. The RRLs are responsible for the training, hand holding and smooth functioning of the CRSs. The staff of the CRS were asked if they faced any challenges with the RRLs with regard to training, supply of packaging materials, testing results being shared by the RRLs etc.

It was observed that all CRSs had received orientation training at the beginning of the study. These trainings were frequent in the initial days of project implementation, and it was later on restricted to when new staff were recruited at many sites. However, most of the CRS said that the project has been running for many years and everybody is familiar with the project requirements and only when staff changes there comes a need for re-training. The NIE team responsible for data management was found to provide regular handholding of these sites regarding data entry in ReDCap. It was observed that laboratory training of RRLs staff was provided by the NAL at least once a year. Some of the CRSs staff did suggest that it would be good to get refresher trainings more often.

*“Before Covid we were getting orientation twice a year. After Covid it’s once a year. From the RRL site we only get oriented once at the beginning of the project. It is fine until the research staff get changed.” CRS PI, Additional Professor in the Department of Paediatrics.*

With regard to the reporting of the test results by RRLs to the CRSs, there were mixed responses. Many of CRSs stated that RRLs did update them regularly about the results, while many stated that there was a delay and results were reported very late even after the patient was discharged from the hospitals. Hence, the laboratory assays aided mostly in surveillance and did not contribute much to diagnosis and routine patient care. As stated by *CRS PI, Assistant Professor from Department of Paediatrics:*

*“The results were made available very late. If we get results in regular intervals or at shorter intervals, we can release the results to our units and update them if the child was infected with Streptococcus pneumoniae. That child can be called for a follow up and can be monitored better. Ultimately the purpose is to identify the organism and to prevent the morbidity.”*

Sites did not face much issues regarding the sample packaging and supply of materials for the same. Most of the CRSs were provided materials on a regular basis by RRLs. In case of delay of delivery of packaging materials, it was observed that the CRSs used materials that are available in their medical colleges, or they bought it locally and it was later reimbursed by the RRLs.

### Challenges with data management team

The data management system for this large surveillance network was maintained centrally at the ICMR NIE which was also an RRL. To ensure data quality and to minimize data errors, data was collected initially on hard copies of the CRF’s. These were in the form of a booklet that was being provided by the data management center at NIE. The research associates entered the data into the hard copies which was then entered into the ReDCap database. The hard copies were then shipped back to NIE, Chennai, where the data mangers cross checked the data in the hardcopies with data entered in the ReDCap. If there were any queries, the data manager would call and resolve it with the research assistant before locking the data in the database. Even though this was found to be time consuming in nature, this process of double checking enabled to reduce data entry errors. When enquired about the challenges the sites faced with data entry most of them stated that there were periods when there was a shortage of the hardcopies of the CRF’s. During which time the sites had to photocopy the forms and enter the data in it. The costs were then reimbursed to the site.

*“As we do not have CRF form we used to photocopy our form, fill it, then again send them to photocopy, so it will take about 2–3 days to do the whole process” Technical Assistant, CRS.*

All sites were satisfied by the support provided by the data management team, they said that orientation and refresher trainings were regularly being provided to them. They were also easily accessible for clarification of doubts. WhatsApp groups were also formed for easy communication which resulted in prompt clarification of doubts.

*“They are helpful. Whenever we have doubt they clear it. They have created a WhatsApp group with each site, so whenever there is a doubt or when we need clarification, we just post a message in the group and asked for the clarification, it will be done on the same day or next” CRS PI, Assistant Professor in Department of Paediatrics, CRS.*

### Inter-departmental coordination challenges

For the implementation of such a large surveillance network across the country, it needed a lot of coordination within different departments of a tertiary care center and between the different centers spread across the country. Within a tertiary care center different departments like paediatrics, pediatric neurology & pulmonology were all involved from where study participants were identified, and CSF samples were collected. At the pediatric department, it was the responsibility of the junior/ senior residents who were trained by the co-investigator from paediatrics for identifying the study participants and to collect the CSF samples. These samples were then sent to the microbiology department where it was processed, stored and shipped to the RRL. The research associate who was posted in the microbiology department was responsible for coordinating these activities and for collecting the data from the case sheets and for entering it into the CRF’s. Often these departments are situated in different buildings within a medical college campus making it difficult for the investigators to meet in person. Irrespective of these challenges, it was seen that the investigators were able to coordinate among themselves for the smooth implementation of the study. When asked about the common challenges faced, some stated that the hospitals were huge and the departments were situated far away from each other. As one participant stated:

*“It is difficult for us because it is a big hospital and roaming here and there in search of patients is difficult so the coordination between microbiology and the Paediatric department is mandatory.” CRS PI, Professor and Head of the department of Microbiology.*

Transfer of investigators and constant rotation of junior/ senior residents were also identified as major challenges. Every time the investigators changed, or the residents rotated, orientation sessions needed to be conducted.

*“PI of the Paediatric Department was transferred to some other medical college, so we had to again coordinate with the new PI of the Department.” CRS PI, Professor and Head in the department of Microbiology.*

In some colleges, it was observed that only 50% of the residents rotated at a given point of time. This resulted in some of the experienced residents being available for handholding the newer residents.

*“We have a good rotation system. We do not rotate all but only 50% of them. Either JRs or SRs.” CRS Co-PI, Addl Professor, Paediatrics Department.*

Even if the interns and residents rotate, all hospitals regularly conducted training sessions in order to orient the newcomers.

*“Every month there is a change of intern and after every 4 months there is a change in PG students. So every 4 months we used to explain to them about this project like what are the samples we are supposed to collect, from whom we are supposed to collect. We trained the PGs and they in turn train the new interns.” CRS PI, Microbiologist.*

*“If any particular resident is forgetting to send the sample, we individually call that resident, so it is an ongoing system. Whenever there is any roadblock, we try to remove it as soon as possible.” RRL PI, Professor and HOD of Paediatrics.*

For ensuring smooth communication between the different departments, some of them even formed WhatsApp groups where regular updates of patients admitted were posted, in-order to ensure that no one was missed out.

*“We have made a group on WhatsApp where JRs, SRs post about patients being admitted in their wards so that they may be contacted for participation in the study. This way we ensured that we did not miss anybody” Research Assistant, Paediatric Department.*

Hence, by timely orientation and setting up of easy communication channels, the departments were able to overcome the interdepartmental coordination challenges.

### Areas of improvement

Each of the investigators was asked if they had any specific suggestions for improving the quality of the research project and some of them stated that the role played by the CRS in the project needs to be increased. They were of the opinion that currently the CRS sites were functioning only as centers for sample collection and were not carrying out laboratory analysis which they felt they were equipped to do. They felt that the PCR should be conducted at the CRS level so that results could be reported early enough for providing appropriate clinical treatment, while the RRLs could do the serotyping on the samples. As some of the site PIs stated:

“*Involvement of CRS should be more and should not only be restricted to sample collection. Some more tasks should be given for the CRS since this will motivate them. Currently the CRS PI’s involvement is very minimal. If more involvement is there for the CRS, then PI will be more motivated, and they will do,” CRS PI, Professor and Head in the department of Microbiology.*

*“If PCR can be started over here at the CRS, we will be able to get the results in time and we would be able to give the appropriate treatment on time.” Research Assistant, CRS.*

One of the other suggestions made by the sites was to start satellite sites near their vicinity in order to increase the enrolment of more participants in the study. While some of the others felt that instead of starting new sites, it would be best to improve the coordination between the microbiology and paediatrics departments in order to ensure that cases are not missed out due to communication gaps.

*“We can collaborate with big hospitals and nursing homes and ask them to be satellite sites, because they are admitting a large number of cases*.” *Co PI CRS, Additional Professor of the Department of Paediatrics.*

*“There should be more coordination between the PIs of both Microbiology and Paediatric Department so that no cases get missed.” PI RRL, Professor and Head in the Department of Microbiology.*

Other than these suggestions, the sites also highlighted the need for prompt transportation of the samples from the CRS to the labs followed by immediate analysis of the samples. It was observed that samples were collected and stored at the CRS for a long time before they were shipped and analyzed at the labs resulting in reduced sample yield. Long duration of storage was seen to hamper the quality of the samples resulting in false negative results. Most sites waited for accumulation of samples in-order to decrease the cost of courier charges, thereby affecting the quality and timeliness of results.

*“Storage and transportation are equally important. Sometimes transportation gets delayed. We try to send samples weekly when there are enough samples instead of sending them fortnightly or once in a month. Coordination, proper collection, proper storage, proper transportation are the things that can improve the yield. At every point we have to take care.” PI CRS, Professor and Head in the Department of Microbiology.*

*“Quick turnover of the CSF samples so that at least CSF samples can be shipped once monthly or every 15 days and if the samples are processed faster, the reports can be available in time for treatment and follow up of the patients.” Co-PI CRS, Assistant Professor in Department of Paediatrics.*

One of the investigators also highlighted the need for following up the participants after they were discharged from the hospital or after they left against medical advice (LAMA). Since this was a cross-sectional study, the actual outcomes of these children were not captured. Data about their treatment outcomes, sequelae following meningitis infection or negative outcomes such as death were not being captured. It was suggested that a subset of these patients may be followed up to capture their outcomes. It would be interesting to note the outcomes of the LAMA cases to see what happened to them as well. It was also suggested to look if any of the children suffered from any neurological sequel as a result of meningitis.

*“Follow up of the cases is important. What has happened to those patients who got treated and discharged we actually tried to do it from our side in our hospital. Like in the short term, what would happen if the patient was discharged without sequelae? have they gone into relapse? Just to know their follow up status and understand whether they suffered from any sequelae. So recording those data might help to know how these meningitis cases have progressed” Scientist, CRS.*

When asked if this project needs to be extended, all investigators uniformly agreed that the project is very relevant, and this data is crucial for deciding on the vaccine strain that needs to be incorporated into the UIP schedule. All of them stated that this project should not end and instead it should be integrated into the routine surveillance network of the country. As one PI stated:

*“I am satisfied that we are generating this data and this aids in improving patient care and also contributes to decision making of the types of strains to be included in the vaccine. We have to see which serotype is prevalent in our part of the country, vaccine strain of pneumococcal vaccine, whether the current vaccine is sufficient or we have to change. All these questions can be answered only if surveillance is ongoing.” CRS PI, HOD Paediatrics.*

The challenges identified in this paper did affect some of the surveillance performance indicators especially in the initial stages of the implementation of the laboratory surveillance network. Enrolment rates of under five children with suspected meningitis in the study were initially low due to unwillingness of parents to give consent for CSF collection. However, over time study enrolments improved when the investigators spend time with the parents to clarify their concerns. Cases enrolled initially was also low because of the difficulty in case identification due to broad WHO case definition and this indicator improved overtime with repeated refresher training sessions for staff members. Inadequate CSF sample collection both in quality and quantity also affected the surveillance performance. Hence root cause analysis and corrective & preventive actions in the form of trainings were provided to the staff involved in the collection, storage and transportation of samples. Therefore the challenges in patient identification, enrolment, sample collection and testing all did affect the surveillance performance in the initial days of establishment of this nationwide network, however over time through effective monitoring and supportive supervision the network has been established and is capturing valuable data that aids evidence based decision making.

Even though setting up this huge surveillance network across the country by involving multiple partners was quite challenging, it was found to be rewarding at the same time. This is the only national level meningitis surveillance network that monitors the circulating PCV serotypes in the country. Hence, it is crucial to continue this surveillance in a sustainable manner by integrating it into the routine national surveillance systems existing in the country.

## Discussion

This study was able to identify the challenges and facilitators faced in the implementation of a nationwide Laboratory Surveillance network for Pneumococcal Meningitis in India. The lessons learnt from this study will be useful for consideration while planning the execution of future such surveillance networks in the country as well as other countries.

The key to the success of any surveillance system is the use of an appropriate case definition that is sensitive enough to identify the study participants so that no one gets left out and at the same time it is not too broad to lose its specificity. As per WHO, case definitions are needed to ensure that surveillance data are comparable between countries. Also, case definitions have a crucial impact on the sensitivity and the positive predictive value of a surveillance system. In this project too, the WHO definition was used for meningitis surveillance which included numerous symptoms such as fever, neck stiffness, bulging fontanelle, altered or reduced level of consciousness, prostration, lethargy, convulsions and any clinical suspicion of meningitis by the physician (12, 13). Majority of the medical officers felt this definition to be very broad and lacked specificity. They felt that most often, eliciting the history of prostration and lethargy from the parents was difficult. Hence the definition needs to be made more specific.

One of the reasons for the successful implementation of this network was the appropriate selection of sentinel sites which had dedicated investigators who adhered to the protocol to recruit study participants. The WHO interim global epidemiological surveillance standards also highlight that system stability, feasibility and representativeness are the most important factors to be considered while choosing a sentinel site. The guideline also goes on to state that when multiple sentinel sites are being considered, sites should represent diverse populations or climate zones since this will provide information about transmission patterns among sub-populations with unique demographic or socio-economic characteristics. The guideline also states that there is no ideal number of sentinel sites in a country but that countries should start with one or a few sentinel sites and only expand if they function well (14). The site selections and implementation for this surveillance network too was conducted taking into consideration these factors and network expanded slowly in a phased manner across various geographies of the country.

The other major step toward participant enrolment was to get informed consent for data collection and CSF sample collection in order to capture the different circulating serotypes in the country. However, obtaining consent and retrieval of proper clinical history were difficult since most of them were from rural areas and were illiterate. Since the parents often rushed to these centers with very sick children, they mostly did not carry any discharge history or details of the medicines given to the child, thereby making it difficult to understand if antibiotics were provided prior to the current hospital visit or not. The parents also did not carry the vaccination cards with them making vaccination history retrieval very difficult. The children were mostly very sick and dehydrated at the time of reporting resulting in a dry tap or low volume of CSF making CSF analysis challenging. In order to overcome these challenges, the investigators had repeated discussions with the parents to gather information and convince them about giving consent for CSF taping. The investigators also reached out after the child got discharged to get the vaccination data through WhatsApp and also by contacting the local health care workers. However this still remained a major challenge in the implementation of the surveillance network. Going forward, these challenges may be overcome by the new Government of India initiatives that are under process such as the Integrated Child Health Record (ICHR) (15) and the U-WIN Portal for digitalization of medical records for children which are gaining momentum (16). These initiatives aim to streamline and improve healthcare for children by leveraging technology to create secure, accessible digital records, offering benefits like real-time access to medical history, vaccination schedules, and growth milestones. The U-WIN Portal, launched in October 2024, is developed for the complete digitization of vaccination services, and maintaining vaccination records for pregnant women and children from birth to 17 years under the Universal Immunization Program. The citizen-centric services of the digital platform include ‘Anytime Access’ and ‘Anywhere’ vaccination services, self-registration by citizens using the U-WIN web-portal or the U-WIN citizen mobile application, automated SMS alerts, universal QR-based e-Vaccination Certificate and utility to create their Ayushman Bharat Health Account (ABHA) ID for themselves and child ABHA ID for their children (17). Therefore going forward, the ICHR and U-WIN platforms may be utilized for the follow up the medical records and vaccination data of the children.

Another crucial step in setting up a national laboratory surveillance network by the creation of a hierarchical system of laboratories starting with a basic laboratory at the periphery from where samples are collected and basic tests are done for patient management. Followed by further processing of samples, storage and transportation to higher laboratories for serotyping and analysis. Since, this network only included tertiary care centers, basic laboratory facilities were all available at all the CRSs and RRLs facilities hence it did not require any additional investments. The National apex laboratories selected for the study were one of the best in the country, however they required support for the establishment of laboratory assays for TAQMAN based serotyping for which technical support and lab support in the assay kits, reagents, primers & probes were being provided by the external funders and agencies. However, this was not found to be a major lacunae of this surveillance network, because the project depended on external support. Therefore, for the establishment of a sustainable lab surveillance network it is important that diagnostic tests, reagents, primers and probes are all manufactured indigenously and not being dependent on external agencies. Hence, for the establishment of a successful model it is crucial that it should be self-sufficient and sustainable. Even though this network was established on external funding and technical support, going forward it is planned to be merged with the routine surveillance network of Infectious Disease Research and Diagnostic Laboratory (IRDLs) (18)whose data is fed into to the Integrated Health Information Platform (IHIP). These IRDLs are fully supported and funded by the Department of Health Research, Ministry of Health & Family Welfare, Government of India (19). Additionally, training and capacity building sessions are being undertaken for laboratories to conduct PCV serotyping and efforts are being made to make this lab surveillance network self-sustainable by providing in-house lab materials.

Similar successful implementation of laboratory surveillance for bacterial meningitis have been documented in Vietnam and Ghana. The Vietnam Meningitis Encephalitis Surveillance Network was established in 1998 and it was implemented in two hospitals in Ho Chi Minh City to monitor bacterial meningitis in children under five. This network was supported by the National Institute of Hygiene and Epidemiology, the World Health Organization (WHO), and the US Centers for Disease Control and Prevention Representative Office in Vietnam which provided both financial and technical support (20). Similarly in Ghana, in 2008, WHO set up the Global Invasive Bacterial Vaccine-preventable Diseases surveillance network that focussed on meningitis surveillance at 2 sentinel sites (21). From these documented evidences, it is evident that in the initial stages of implementation of such nationwide laboratory surveillance networks Low and Middle-Income Countries (LMICs) often require substantial support from global health agencies such as the WHO, along with specialized technical assistance from experienced international partners for establishing robust sentinel surveillance systems. During the initial phases, external collaborators play a critical role in capacity building, infrastructure development, training of local personnel, and establishing standardized surveillance protocols and data management frameworks. However, to ensure long-term sustainability and national ownership, it is essential that these systems gradually transition to local leadership and financing. Over time, external support should intentionally be phased out, enabling the country to independently manage, maintain, and scale the surveillance network as part of routine public health operations.

Two other crucial requirements for establishing a surveillance network is the need for a data management team and a central project coordination unit (22). In this project, the data management team was hosted at the ICMR NIE where the data collected on the ReDCap database from this entire network was stored in a separate server. This team consisted of experienced laboratory experts, epidemiologists and statisticians who are continuously reviewing the data, carrying out statistical analysis and reviewing the circulating PCV strains in the country. Last but not the least, for the smooth implementation and functioning of such a large surveillance network there needs to be a central coordination unit who is responsible for coordinating with the NALs, RRLs, CRSs, NIE and external agencies for the implementation of the project. This unit is responsible for fund release, coordination of the activities under the project, capacity building activities and supervision of data management activities. Therefore, while setting up such a surveillance system, these two components are also crucial components along with the lab network. The strengths of this evaluation study was that it was able to document the implementation of the network across all the tiers and all stakeholders. However, the study did not capture the perspectives of the funders and the CDC laboratory technical team. Other limitations of the study was that this was a qualitative study using purposive sampling thereby making the findings context specific with limited generalizability to other sites. This evaluation was conducted by study coordinators at the ICMR Headquarters, thereby resulting in potential study respondent bias. However, all efforts were made to create trust among the respondents for sharing the ground realities they faced while implementing the surveillance network. Additionally, data and researcher triangulation methods enabled to increase the validity and reliability of the study findings. Since, study participants were trained medical / research staff, the interviews were all conducted in English and was transcribed verbatim, no data was lost due to translation inconsistences.

## Conclusion

This nationwide laboratory Surveillance for Pneumococcal Meningitis was implemented in the year 2018 with the aim to evaluate the impact of the PCV rollout on pneumococcal disease burden and to gather comprehensive data on circulating *Streptococcus pneumoniae* serotypes across the country. Since, this network covered most part of the country, the data from this study is being routinely reviewed and used by the National Technical Advisory Group on Immunization of India for making policy decisions regarding vaccine strain change. Therefore this network was found to be successfully implemented because it was providing valuable evidence for decision making by the policy makers.

Therefore, the implementation of this nationwide laboratory surveillance across the country has been successful irrespective of the many challenges it faced. This network has been immensely useful for gathering evidence for making policy decisions regarding the use of PCV. This is a classic example of a successful externally funded project that has now transitioned into an in-country sustainable model. The lessons learnt from this study have been documented so that this learning can be replicated by others who are planning to implement such surveillance networks in their countries. As per our experience, it is important to have a decentralized system of sentinel laboratory surveillance network with an inbuilt data management system and central coordination unit to oversee the activities of the network. Even if external technical and funding support is taken for the establishment of such networks, it is very important to plan and prepare beforehand a sustainable plan of transition and integration into the country’s surveillance system. If not, it would result in the demolition of the network once the funding retracts. Based on our experiences, we strongly recommend that every country establish a national laboratory surveillance networks to monitor the circulating pneumococcal serotypes and other high burden vaccine preventable serotypes so that evidence-based decisions can be made regarding the type of vaccines that are being used in a country.

## Data Availability

The raw data supporting the conclusions of this article will be made available by the authors, without undue reservation.

## Group members of the Meningitis surveillance group

Adil Raza, Jawaharlal Nehru Medical College, Aligarh, India; Ajanta Sharma, Gauhati Medical College and Hospital, Gauhati, India; Alok Kumar Deb, ICMR-National Institute of Bacterial Infections, Kolkata, India; Amita Jain, King George’s Medical University, Lucknow, India; Amit Garg, Lala Lajpat Rai Memorial Medical College, Meerut, India; Anil Kumar Chaudhary, Rajendra Institute of Medical Sciences, Ranchi, India; Anil Kumar Tiwari, Patna Medical College and Hospital, Patna, India; Anubha Srivastava, Moti Lal Nehru Medical College, Allahabad, India; Aparna Alladi, Kakatiya Medical College, Warangal, India; Aparna Parmar, Pt. B. D. Sharma University of Health Sciences, Rohtak, India; Arunabha Sarkar, North Bengal Medical College, Siliguri, India; Ashwin Belludi, National Institute of Mental Health and Neurosciences, Bengaluru, India; Ashok Kumar, Darbhanga Medical College and Hospital, Darbhanga, India; Balaji Veeraraghavan, Christian Medical College, Vellore, India; Balasubramanian Sundaram, Kanchi Kamakoti CHILDS Trust Hospital, Chennai, India; Bhadresh R Vyas, M. P. Shah Government Medical College and Hospital, Jamnagar, India; Bhagat Baghel, Late Baliram Kashyap Memorial Government Medical College, Jagdalpur, India; Bharti Malhotra, Sawai Man Singh Medical College, Jaipur, India; Bhavana Jagannatha, Indira Gandhi Institute of Child Health, Bengaluru, India; Dilip Turbadkar, Lokmanya Tilak Municipal Medical College and General Hospital, Mumbai, India; Ghanshyam Saini, Government Medical College, Jammu, India; Girish Kumar Chethrapilly Purushothaman, ICMR-National Institute of Epidemiology, Chennai, India; Kalpana Datta, Medical College, Kolkata, India; Kuldeep Singh, All India Institute of Medical Sciences, Jodhpur, India; Milap Sharma, Dr. Rajendra Prasad Government Medical College, Tanda, India; Narender Pal Singh, University College of Medical Sciences, Delhi, India; Nirbhay Mehta, Mahatma Gandhi Memorial Medical College, Indore, India; Nowneet Kumar Bhat, All India Institute of Medical Sciences, Rishikesh, India; Poovazhagi Varadarajan, Institute of Child Health and Hospital for Children, Madras Medical College, Chennai, India; Pragalatha Kumar, Indira Gandhi Institute of Child Health, Bengaluru, India; Priya Sreenivasan, Government Medical College, Thiruvananthapuram, India; Purbasha Ghosh, Burdwan Medical College, Burdwan, India; Rakesh Sharma, Indira Gandhi Medical College, Shimla, India; Reema Nath, Assam Medical College, Dibrugarh, India; Sandip Samanta, Dr. B C Roy Post Graduate Institute of Paediatric Sciences, Kolkata, India; Shikha Malik, All India Institute of Medical Sciences, Bhopal, India; Suman Kanungo, ICMR-National Institute of Bacterial Infections, Kolkata, India; Veeraraja Basavanthappa Sathenahalli, Indira Gandhi Institute of Child Health, Bengaluru, India; Vijay Kumar Guduru, Kakatiya Medical College, Warangal, India; Vishal Guglani, Government Medical College and Hospital, Chandigarh, India.
